# Artificial neural networks for simultaneously predicting the risk of multiple co‐occurring symptoms among patients with cancer

**DOI:** 10.1002/cam4.3685

**Published:** 2020-12-22

**Authors:** Wenhui Xuyi, Hsien Seow, Rinku Sutradhar

**Affiliations:** ^1^ Division of Biostatistics Dalla Lana School of Public Health University of Toronto Toronto ON USA; ^2^ ICES Toronto ON USA; ^3^ Department of Oncology McMaster University Hamilton ON Canada; ^4^ Institute of Health Policy, Management and Evaluation University of Toronto Toronto ON USA

**Keywords:** artificial neural network, calibration, co‐occurrence, discrimination, Edmonton Symptom Assessment System, model validation, simultaneous prediction, symptom burden

## Abstract

Patients with cancer often exhibit multiple co‐occurring symptoms which can impact the type of treatment received, recovery, and long‐term health. We aim to simultaneously predict the risk of three symptoms: severe pain, moderate‐severe depression, and poor well‐being in order to flag patients who may benefit from pre‐emptive early symptom management. This was a retrospective population‐based cohort study of adults diagnosed with cancer between 2008 and 2015. We developed and tested an Artificial Neural Network (ANN) model to predict the risk of multiple co‐occurring symptoms within 6 months after diagnosis. The ANN model derived from a training cohort was assessed on an independent test cohort for model performance based on sensitivity, specificity, accuracy, AUC, and calibration. The mutually exclusive training and test cohorts consisted of 35,606 and 10,498 patients, respectively. The area under the curve for the risk of experiencing severe pain, moderate‐severe depression, and poor well‐being were 71%, 73%, and 70%, respectively. Patient characteristics at highest risk of simultaneously experiencing these three symptoms included: those with lung cancer, late stage cancer, existing chronic conditions such as osteoarthritis, mood disorder, hypertension, diabetes, and coronary disease. Patients with over a 40% risk of severe pain also had over a 70% risk of depression, and over a 55% risk of poor well‐being. Our ANN model was able to simultaneously predict the risk of pain, depression, and lack of well‐being. Accurate prediction of future symptom burden can serve as an early indicator tool so that providers can implement timely interventions for symptom management, ultimately improving cancer care and quality of life.

## Graphical abstract

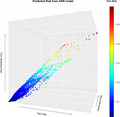

## 1 INTRODUCTION

### 1.1 Clinical background

Patients diagnosed with cancer may experience multiple symptoms, often co‐occurring throughout their disease trajectory. Commonly reported symptoms include pain, fatigue, anxiety, lack of appetite, and lack of well‐being^1^, ^2^, ^3^, ^4^; and a majority of patients experience both severe fatigue and lack of well‐being in the initial months after diagnosis.^5^ The prevalence and intensity of these symptoms varies by patient characteristics. Younger age, female sex, higher comorbidity burden, and lower income have been shown to be associated with an increased likelihood of experiencing severe symptoms.^5^ Severe symptoms can have an impact on the type of treatment received, on recovery and on long‐term health outcomes.^2^ It is, therefore, important for patients to receive timely and appropriate symptom management throughout their cancer trajectory. Being able to simultaneously predict the risk of experiencing multiple co‐occurring symptoms based on a patient’s profile and characteristics can assist cancer care providers in developing a plan for timely symptom management. Such risk prediction tools can offer opportunities for intervention so that patients experience less symptom distress or are better prepared for symptom exacerbations.

### 1.2 Methodological background

The majority of studies examining predictors and risk of symptom burden among patients with cancer utilize traditional statistical techniques, such as logistic or modified Poisson regression.^5^, ^6^, ^7^ When several symptoms for each patient are being considered, each symptom is often examined separately meaning that an independent regression model is developed for each symptom. Unfortunately, this approach fails to account for the possibly strong correlation in symptom burden measures taken from the same patient.^8^ Moreover, the relationship between patient‐level predictors and symptom burden may be nonlinear and complex, making it difficult to explicitly capture using traditional regression techniques.

As machine learning techniques are gaining attention as prediction tools in health research, it is of interest to determine if they can be used to simultaneously predict multiple outcomes of symptom burden among patients with cancer. An Artificial Neural Network (ANN) offers a convenient way to use large volumes of individual‐level data to predict multiple co‐occurring outcomes. The basic ANN structure consists of three layers: an input layer, a hidden layer, and an output layer. The patient‐level predictors are represented as nodes in the input layer, and the patient‐level outcomes are represented as nodes in the output layer. The nodes in the hidden layer are intermediate unobserved values that allow the ANN to model complex nonlinear relationships between the input nodes and the output nodes.^9^, ^10^ To address gaps in prior approaches that predict symptom burden risk, we sought to develop and validate an Artificial Neural Network to *simultaneously predict* the risk of experiencing *multiple co‐occurring symptoms* among patients with cancer.

## 2 METHODS

### 2.1 Study design and population

This was a population‐based retrospective cohort study among all adults diagnosed with cancer in Ontario, Canada, during 2008–2015. Individuals without a valid health card and those that did not participate in symptom screening were excluded from the cohort.

### 2.2 Data sources

In Ontario, Canada’s most populous province, the Edmonton Symptom Assessment System is a validated tool implemented across cancer centers to screen for nine common symptoms. The severity at the time of assessment of each symptom is rated from 0 to 10 on a numerical scale; with 0 meaning that the symptom is absent and 10 that it is the worst possible severity.^11^, ^12^ For cancer patients in Ontario receiving home care or residing in a long‐term care facility, the interRAI Assessment System is another validated set of tools that also screens for symptoms such as pain and depression, and can provide valuable information to support person‐specific care planning across the continuum of care.^13^

We used the following linked administrative databases [to retrieve the corresponding information]: (a) Ontario Cancer Registry [diagnosis date, cancer type, cancer stage]; (b) Registered Persons Database [age, sex, death date, postal code]; (c) Activity Level Reporting database [chemotherapy regime, radiation treatment]; (d) Discharge Abstract Database [hospitalization dates and reasons, cancer surgery, comorbidities]; (e) National Acute Care Registry System database [emergency department visit dates and reasons]; (f) Ontario Health Insurance Plan database [physician visits, physician billing codes]; (g) Home care database [nursing and/or personal support care visit dates]; (h) Symptom Management Reporting Database / ESAS database [symptom burden, performance status]; and (i) interRAI databases [symptom burden, performance status]. All administrative databases were linked using unique encoded identifiers and analyzed at ICES (historically known as the Institute for Clinical Evaluative Sciences).

### 2.3 Outcomes (ANN output nodes)

A priori, we were interested in predicting the presence of three specific symptoms over a 6‐month window (within 3–9 months after a cancer diagnosis): severe pain, moderate to severe depression, and poor well‐being, respectively, representing physical, psychosocial, and global symptom measures. For every patient, information on the presence/absence of each of these three symptoms was retrieved in a hierarchical manner from the Symptom Management Reporting Database and the interRAI databases, similar to our prior work^7^:

1. Severe pain: Defined as a score of 7–10 (severe) for pain on ESAS; or a score of 3 (severe or excruciating) for pain intensity from the interRAI
2. Moderate‐severe depression: Defined as a score of 4–10 (moderate to severe) for depression on ESAS; or a score of 3 or more on the Depression Rating Scale from the interRAI
3. Poor well‐being: Defined as a score of 7–10 (poor) well‐being on ESAS; or Yes for “client feels he/she has poor health when asked” under health status indicators from the interRAI.

### 2.4 Covariates (ANN input nodes)

The following 39 unique covariates were measured on each patient (within the first 3 months after diagnosis):

1. Demographic characteristics (determined at diagnosis): age (continuous), sex (binary), distance from cancer center within 50 km (binary);
2. Clinical characteristics (determined at diagnosis): cancer type (categorical), cancer stage (categorical), presence of one of 17 other chronic diseases as determined by validated algorithms^2425^ (17 binary indicators);
3. Treatment characteristics (determined within 3 months after diagnosis): receipt of chemotherapy (binary), radiation treatment (binary), cancer surgery (binary);
4. Baseline patient‐reported measures (determined within 3 months after diagnosis): Performance status (categorical), symptom burden status (categorical) for each of nine symptoms;
5. Health care utilization measures (determined within 3 months after diagnosis): has a primary care physician (binary), hospitalization (binary), has a live‐in caregiver (binary), receipt of end‐of‐life homecare services (binary).

It should be noted that the exposure measurement window (within 3 months after diagnosis) was distinct from the outcome measurement window (3–9 months after diagnosis).

### 2.5 Statistical analyses

#### 2.5.1 Descriptive analyses

Prior to initiating any modeling, we randomly divided our population into two mutually exclusive cohorts: 75% of patients comprised the training cohort and the remaining 25% of patients comprised the test cohort. The distributions of characteristics for both training and test cohorts were explored; continuous measures were described with medians and interquartile ranges, and categorical measures were described using frequencies and percentages.

#### 2.5.2 Artificial Neural Network Model for simultaneously predicting pain, depression, and well‐being

**FIGURE 1:**
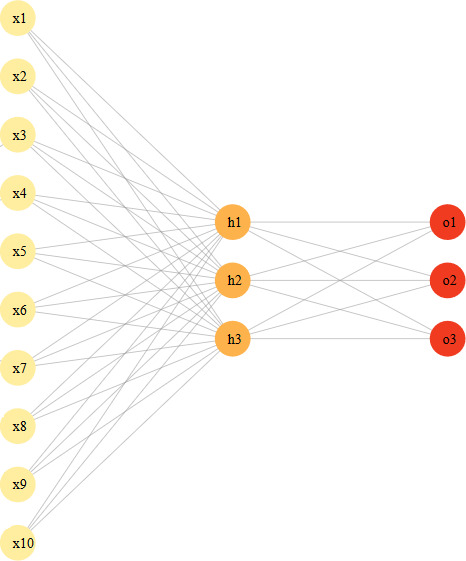
Visualization of a 10‐3‐3 neural network: 1 input layer consisting of 10 nodes (x1–x10), 1 hidden layer consisting of 3 nodes (h1–h3), and 1 output layer consisting of 3 nodes (o1–o3). The grey lines represent the connections/weights that need to be estimated. Note the network described herein was 39‐3‐3

We developed both 3‐layer and 4‐layer perceptron models. The 3‐layer network consisted of an input layer, 1 hidden layer, and an output layer; the 4‐layer network consisted of an input layer, 2 hidden layers, and an output layer. All 39 covariates described above were represented as nodes in the input layer, which were normalized and encoded as required.^14^ The output layer consisted of three nodes representing each of our binary symptom outcomes (severe pain yes/no, moderate to severe depression yes/no, poor well‐being yes/no). We began with a 3‐layer model with two nodes in the first hidden layer, after which 1 additional node was incorporated until we reached 10 nodes in the first hidden layer. This process was repeated with a 4‐layer model that included two nodes in its second hidden layer. The weights of the neural network were estimated using the training cohort. This was done using backpropagation with a weight backtracking algorithm, where a cross entropy error function was minimized.^15^ The AUC (area under the ROC curve) value was then calculated for the training cohort to understand the degree of discrimination under each ANN model. We found that values for sensitivity, specificity, and AUC in the training cohort were optimal when we had three nodes in the first hidden layer and no nodes in the second hidden layer. As a result, the final ANN model included an input layer, three nodes in its single hidden layer, and three nodes in the output layer (Figure 1). This is in line with prior recommendations stating that one layer of hidden neurons is generally sufficient for classifying noncomplex data.^16^

#### 2.5.3 Assessing predictive ability

The ANN model was used to predict the 6‐month risk of severe pain, moderate‐severe depression, and poor well‐being for each patient in our test cohort. The estimated single set of weights from the ANN model were able to simultaneously predict each of the three symptoms. For each symptom, the predicted number of outcomes was compared to the actual number of outcomes in the test cohort by composing a confusion matrix. In the test cohort under the ANN model, we calculated sensitivity (true positive fraction), specificity (true negative fraction), accuracy (true positive or negative fraction), and discrimination (measured using the AUC value). Additionally, calibration plots for each symptom were constructed under the ANN model using the test cohort. This was done by grouping patients into deciles (10 groups) based on their predicted risk, and then, plotting the observed symptom risk within a decile against the corresponding mean predicted risk within that decile.^17^, ^18^ Points closer to the 45 degree line indicate better calibration. Individuals predicted to be in the highest decile of risk for all three symptoms were identified from the calibration plots. The distribution of baseline characteristics for these individuals were examined to gain a better understanding of highest risk profiles.

#### 2.5.4 3‐Dimensional visualization

After assessing our ANN model’s ability to simultaneously predict all three outcomes of symptom severity, the marginal 6‐month predicted risks for each symptom for every patient were illustrated with a 3‐dimensional scatter plot. The *x*‐, *y*‐, and *z*‐ axis represent the risk of experiencing severe pain, moderate‐severe depression, and poor well‐being, respectively. As risk is a probability, the range of each axis goes from 0.0 to 1.0. Each point on the plot represents a patient from the test cohort, and the *x*‐, *y*‐, and *z*‐ coordinates of the point represent the corresponding risk estimates for each symptom. All analyses and graphs were completed using statistical software R version 3.6.1.^19^

## 3 RESULTS

The study population consisted of 46,104 unique patients, of which 35,606 patients comprised the training cohort and the remaining 10,498 patients comprised the test cohort. Due to the random selection process, the distributions of baseline characteristics were well‐balanced between the training and test cohorts (Table 1, Table A). The most common diagnoses were breast, lung, and colorectal cancers. More than half of the training cohort suffered from hypertension at the time of diagnosis; 41.8% had osteoarthritis, and diabetes was present in 21.9%. Nearly 13% of patients reported experiencing moderate to severe depression near the time of diagnosis.

**TABLE 1:** Distributions of (selected) characteristics at baseline among the training and test cohorts

Variable	Value	Training cohort	Test cohort
	Total	35,606	10,498
Age at diagnosis	Median (IQR)	64 (55–73)	64 (54–73)
Sex	Female	20391 (57.3%)	6027 (57.4%)
Distance from regional cancer center	<=50 km	28322 (79.5%)	8390 (79.9%)
Cancer type	Breast	8638 (24.3%)	2622 (25%)
	Colorectal	4203 (11.8%)	1230 (11.7%)
	Gynecological	2984 (8.4%)	898 (8.6%)
	Head and Neck	1631 (4.6%)	456 (4.3%)
	Hematology	3724 (10.5%)	1141 (10.9%)
	Lung	4354 (12.2%)	1292 (12.3%)
	Other	2790 (7.8%)	812 (7.7%)
	Other Gastrointestinal	2778 (7.8%)	774 (7.4%)
	Other Genitourinary	1342 (3.8%)	360 (3.4%)
	Prostate	3162 (8.9%)	913 (8.7%)
Cancer stage	1	7146 (20.1%)	2250 (21.4%)
	2	8125 (22.8%)	2409 (22.9%)
	3	6653 (18.7%)	1937 (18.5%)
	4	5351 (15%)	1495 (14.2%)
	Unknown	8331 (23.4%)	2407 (22.9%)
Chronic disease present at cancer diagnosis	AMI	131 (0.4%)	41 (0.4%)
	Arrhythmia	2529 (7.1%)	706 (6.7%)
	Asthma	4698 (13.2%)	1380 (13.1%)
	CHF	1948 (5.5%)	529 (5%)
	COPD	3151 (8.8%)	912 (8.7%)
	Coronary	5060 (14.2%)	1465 (14%)
	Dementia	578 (1.6%)	160 (1.5%)
	Diabetes	7802 (21.9%)	2269 (21.6%)
	Hypertension	18456 (51.8%)	5364 (51.1%)
	IBD	222 (0.6%)	75 (0.7%)
	Mental Health	1301 (3.7%)	349 (3.3%)
	Mood Disorder	4461 (12.5%)	1302 (12.4%)
	Osteoarthritis	14887 (41.8%)	4496 (42.8%)
	Osteoporosis	1932 (5.4%)	555 (5.3%)
	Renal Disease	1621 (4.6%)	433 (4.1%)
	Rheumatoid Arthritis	659 (1.9%)	207 (2%)
	Stroke	953 (2.7%)	258 (2.5%)
Chemotherapy within 3 months after diagnosis	Yes	12003 (33.7%)	3538 (33.7%)
Radiation within 3 months after diagnosis	Yes	9475 (26.6%)	2755 (26.2%)
Cancer surgery within 3 months after diagnosis	Yes	17651 (49.6%)	5199 (49.5%)
Has primary care physician	Yes	34790 (97.7%)	10253 (97.7%)
Hospitalization within 3 months after diagnosis	Yes	2187 (6.1%)	648 (6.2%)
Has a live‐in caregiver	No	693 (1.9%)	185 (1.8%)
	Yes	1582 (4.4%)	471 (4.5%)
	Missing or NA	33331 (93.6%)	9842 (93.7%)
Received end‐of‐life care	Yes	1563 (4.4%)	412 (3.9%)

Median and IQR provided for continuous covariates; frequencies and percentages provided for binary or categorical covariates.

Complete table with entire list of covariates can be found in Supplementary Table A.

**FIGURE 2:**
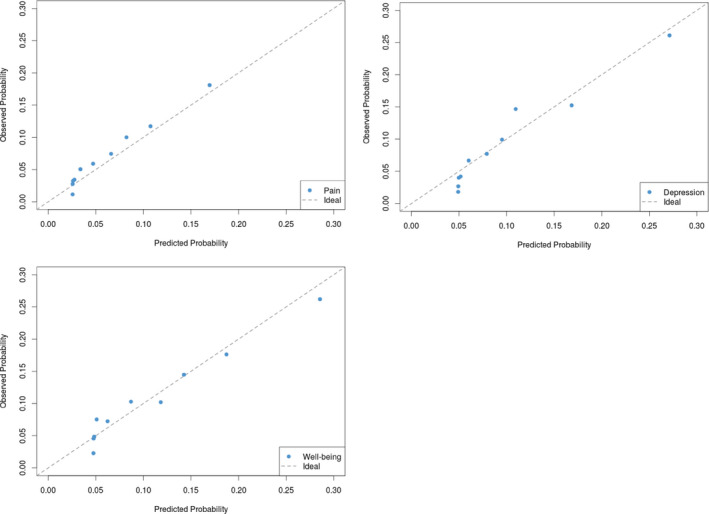
Calibration plot for each symptom under the ANN risk prediction model (on the test cohort)

As mentioned above, several ANN structures with 1 and 2 hidden layers were developed and assessed for simultaneously predicting the 6‐month risk of three symptoms: severe pain, moderate‐severe depression, and poor well‐being. The final ANN model included an input layer, three nodes in its single hidden layer, and three nodes in the output layer (Figure 1). Although this ANN framework jointly models all three symptoms, the marginal risk probabilities of each symptom can still be extracted. Table 2 provides the prediction performance of the ANN model (which was derived from the training cohort) on the test cohort. The marginal estimates of sensitivity, specificity, accuracy, and discrimination are given for each symptom. The area under the curve for the risk of experiencing severe pain, moderate‐severe depression, and poor well‐being were 71%, 73%, and 70%, respectively. The mean marginal predicted risk and the marginal observed risk for each symptom were computed on the test cohort to illustrate calibration (Figure 2). Overall for each symptom, the dots (representing each decile of predicted risk) are tight along the 45 degree line. A greater discrepancy can be seen among patients in the lowest deciles of predicted risk (dots to the left of each plot). The ANN model appears to overestimate the risk, as the mean predicted risk is larger than the observed risk; this finding is consistent for all three symptoms. The ANN model performs specifically well for patients in the highest deciles of predicted risk (dots to the right of the plot). Table 3 provides the distribution of characteristics among all unique patients whose predicted risks lie in the highest deciles for all three symptoms; that is, individuals with the highest risk of jointly experiencing severe pain, and moderate to severe depression, and poor well‐being. Majority of patients were stage 4, and lung or gastrointestinal cancer were the most common diagnoses types. Nearly 60% of patients with the highest risk of symptom burden suffered from osteoarthritis, and 54% were living with hypertension. Mood disorder and diabetes were also common among this highest risk group.

**TABLE 2:** Prediction performance under the ANN model (on the test cohort)

	ANN prediction model			
	Sensitivity	Specificity	Accuracy	AUC
Risk of severe pain	0.61	0.69	0.69	0.71
Risk of moderate‐severe depression	0.69	0.65	0.65	0.73
Risk of poor well‐being	0.63	0.64	0.64	0.70

**TABLE 3:** Distribution of (selected) baseline characteristics for individuals predicted to be in the highest decile of risk for all three symptoms (from the test cohort)

Variable	Value	Highest risk decile for all three symptoms
	Total	995
Age at diagnosis	Mean (SD)	64.68 (13.05)
Sex	Female	585 (58.8%)
Distance from regional cancer center	<=50 km	831 (83.5%)
Cancer type	Breast	96 (9.6%)
	Colorectal	74 (7.4%)
	Gynecological	97 (9.7%)
	Head and Neck	73 (7.3%)
	Hematology	82 (8.2%)
	Lung	298 (29.9%)
	Other	80 (8.0%)
	Other Gastrointestinal	134 (13.5%)
	Other Genitourinary	41 (4.1%)
	Prostate	20 (2.0%)
Cancer stage	1	92 (9.2%)
	2	101 (10.2%)
	3	194 (19.5%)
	4	347 (34.9%)
	Unknown	261 (26.2%)
Chronic disease present at cancer diagnosis	AMI	< 12 (*)
	Arrhythmia	90 (9.0%)
	Asthma	212 (21.3%)
	CHF	100 (10.1%)
	COPD	193 (19.4%)
	Coronary	228 (22.9%)
	Dementia	34 (3.4%)
	Diabetes	337 (33.9%)
	Hypertension	538 (54.1%)
	IBD	< 12 (*)
	Mental Health	109 (11.0%)
	Mood Disorder	318 (32.0%)
	Osteoarthritis	587 (59.0%)
	Osteoporosis	47 (4.7%)
	Renal Disease	87 (8.7%)
	Rheumatoid Arthritis	41 (4.1%)
	Stroke	39 (3.9%)

Mean and standard deviation (SD) provided for continuous covariates; frequencies and percentages provided for binary or categorical covariates; (*) cell frequency suppressed due to small size

**FIGURE 3:**
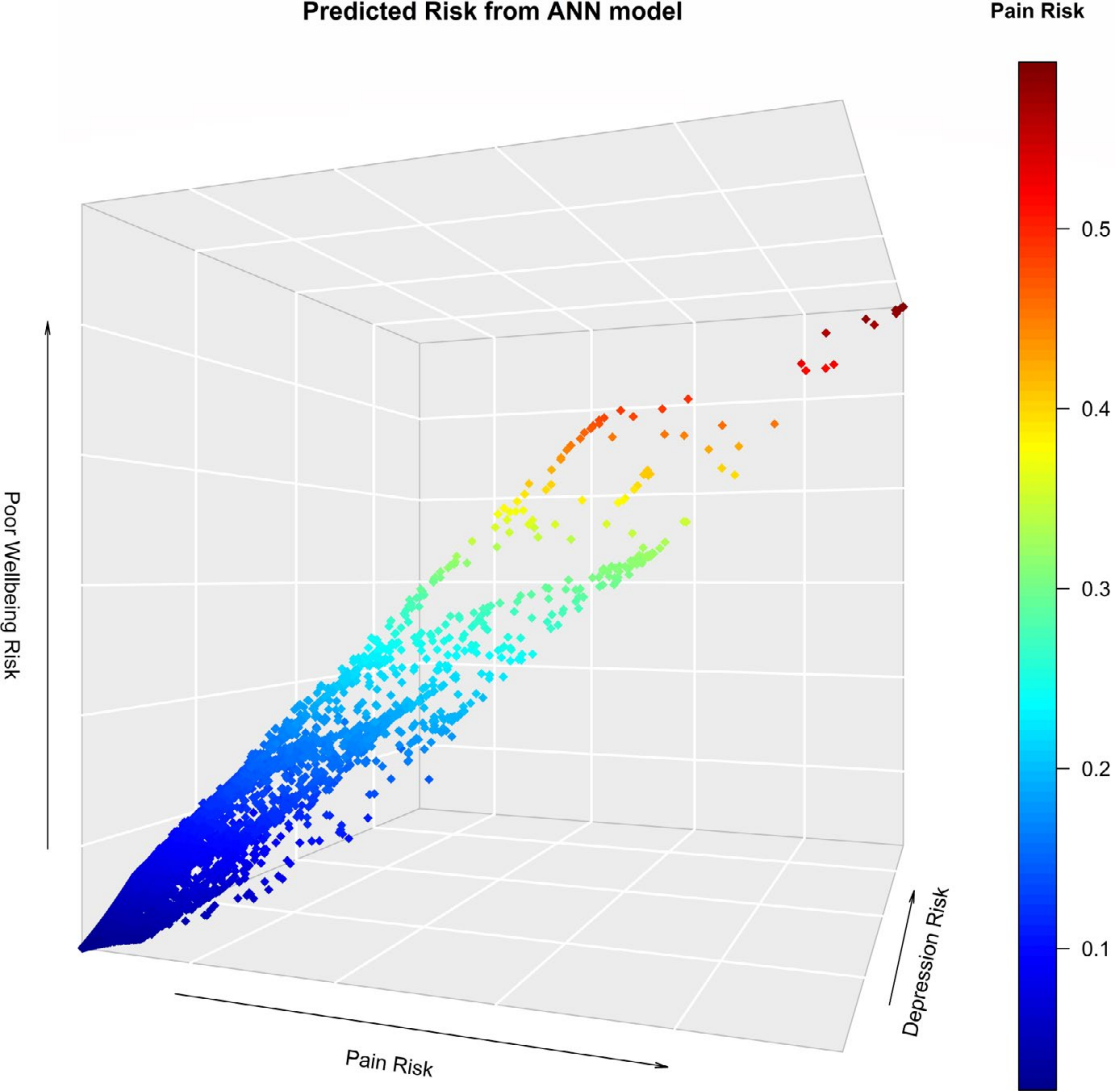
3‐Dimensional scatter plot of predicted risk for each symptom from ANN model (on the test cohort)

Figure 3 provides an illustration of the marginal predicted risks for each symptom for every patient using a 3‐dimensional scatter plot. The plot consists of 10,498 points, one for each patient in the test cohort. There is a clear relationship between pain, depression, and well‐being. As the risk of severe pain and moderate‐severe depression increases, so the does the risk of experiencing poor well‐being. On average, the 6‐month risk of experiencing severe pain was 5.4%, moderate‐severe depression was 8.2%, and lack of well‐being was 7.2%. Patients with over a 40% risk of severe pain also had over a 70% risk of depression, and over a 55% risk of poor well‐being.

## 4 DISCUSSION

The ability to predict the future occurrence of multiple symptoms can be a powerful tool for the cancer care team. Such prediction tools can assist providers in risk‐profiling patients, identifying those at higher risk of symptom burden, and improving the timing of pre‐emptive and personalized symptom management interventions.^20^ The ANN model developed in this paper was able to predict the risk of experiencing three co‐occurring symptom outcomes: pain, depression and lack of well‐being among patients diagnosed with cancer. The model also identified patient characteristics at highest risk of simultaneously experiencing these three symptoms. Profiles including lung cancer, late stage cancer, existing chronic conditions such as osteoarthritis, mood disorder, hypertension, diabetes, and coronary disease can be used to flag patients who may benefit from early symptom management.

ANNs play an important role in risk prediction and can be particularly appealing when aiming to jointly predict multiple outcomes. ANNs are predominantly a distribution‐free data driven approach. These models do not require a priori knowledge on the extent of correlation arising from outcomes within the same individual. In our case, the correlations in the severity of pain, depression, and well‐being were intrinsically captured through the connections in the hidden layers of the network. ANNs also do not require a priori knowledge on the relationships between the predictors and the outcomes, nor do interactions between the predictors need to be prespecified.^17^ Although ANNs are referred to as black‐box models, as estimates of the weights in an ANN cannot be easily interpreted, these models are able to provide individual‐level risk prediction based on a patient’s covariate profile and can be employed as decision support tools once they are integrated into clinical practice.^9^

There has been extensive research conducted on determining factors *associated* with symptom burden among patients with cancer, but very limited work has been done on *predicting* symptom burden.^5^, ^6^, ^21^, ^22^, ^23^, ^24^ A recent study developed a tool using logistic regression for predicting the risk of symptom burden among cancer patients.^7^ Although their model’s level of discrimination was similar compared to our ANN, symptom correlation was ignored and each symptom was predicted independently using separate regressions. Prior work has used machine learning techniques such as support vector regression and nonlinear canonical correlation analysis to predict the severity of multiple co‐occurring symptoms during a cycle of chemotherapy, however, these models were developed and tested with a relatively small sample of cancer patients.^20^

This paper has numerous strengths. To our knowledge, it is the first study to simultaneously predict severity of multiple symptoms under an ANN framework using a population‐based cohort of patients with cancer. With over 46,000 individuals, we were able to build and validate our ANN risk prediction model on large training and test sets, respectively. The model was developed using an extensive list of covariates, including demographic‐, clinical‐, and treatment‐characteristics, and information on baseline patient‐reported measures of functional status and symptom burden, and measures on various types of healthcare utilization. Due to the limited exclusion criteria when creating the province‐wide cohort, our ANN framework, network weight estimates, and findings can likely be applied to other populations of cancer patients receiving universal healthcare.

This study also has several limitations. Information on medications for dealing with symptoms such as pain or depression may improve prediction model performance, however, these data were not available. Symptom screening with ESAS was initiated in Ontario, Canada in 2007, which is the first year of accrual in our cohort. Since uptake of ESAS was gradual across cancer centers, the recent years of data are more representative of the current population of patients participating in symptom screening. We also did not directly compare the risk prediction performance of our ANN model against other commonly used approaches such as logistic regression, or against more complex approaches that account for correlation of multiple outcomes such as joint mixed models. Both techniques require making distributional assumptions, and interactions between predictors and correlation structures for multiple outcomes often need to be explicitly specified. These comparisons remain an important part of our future work with these data.

This study demonstrates the use of ANN models to simultaneously predict the risk of experiencing multiple co‐occurring symptoms among cancer patients. With the growing availability of vast amounts of data on large population‐based cohorts, researchers should consider machine learning techniques particularly when interest lies in predicting several, possible correlated, outcomes.

## 5 ETHICAL STANDARDS

This study involved secondary data analyses only and was thus exempt from requiring REB approval because ICES is a designated “45.1 entity” under the Personal Health Information Protection Act (PHIPA) enabling the use of personal health information.

## CONFLICT OF INTEREST

We declare that we have no conflicts of interest.

## AUTHOR CONTRIBUTION

All authors had roles in design and conduct of the study. WX and RS planned and executed the statistical analyses. All authors had roles in the interpretation of the results, as well as preparation and approval of the Article.

## Supporting information

Supplementary Material

## Data Availability

Research data are not shared.
