# Supplementary material for: Artificial neural networks for simultaneously predicting the risk of multiple co‐occurring symptoms among patients with cancer
Source: Cancer Med. 2020 Dec 22;10(3):989–98. doi: 10.1002/cam4.3685 (PMC7897969; doi:10.1002/cam4.3685)
Supplement: Supplementary file 1 — Supplementary Material [file CAM4-10-989-s001.docx]

**Supplementary Table A.** Distributions of characteristics at baseline among the training and test cohorts

| **Variable** | **Value** | **Training cohort** | **Test cohort** |
| --- | --- | --- | --- |
|  | Total | 35,606 | 10,498 |
| **Age at diagnosis** | Median (IQR) | 64 (55-73) | 64 (54-73) |
| **Sex** | Female | 20391 (57.3%) | 6027 (57.4%) |
| **Distance from regional cancer centre** | <=50 km | 28322 (79.5%) | 8390 (79.9%) |
| **Cancer type** | Breast | 8638 (24.3%) | 2622 (25%) |
|  | Colorectal | 4203 (11.8%) | 1230 (11.7%) |
|  | Gynaecological | 2984 (8.4%) | 898 (8.6%) |
|  | Head and Neck | 1631 (4.6%) | 456 (4.3%) |
|  | Hematology | 3724 (10.5%) | 1141 (10.9%) |
|  | Lung | 4354 (12.2%) | 1292 (12.3%) |
|  | Other | 2790 (7.8%) | 812 (7.7%) |
|  | Other Gastrointestinal | 2778 (7.8%) | 774 (7.4%) |
|  | Other Genitourinary | 1342 (3.8%) | 360 (3.4%) |
|  | Prostate | 3162 (8.9%) | 913 (8.7%) |
| **Cancer stage** | 1 | 7146 (20.1%) | 2250 (21.4%) |
|  | 2 | 8125 (22.8%) | 2409 (22.9%) |
|  | 3 | 6653 (18.7%) | 1937 (18.5%) |
|  | 4 | 5351 (15%) | 1495 (14.2%) |
|  | Unknown | 8331 (23.4%) | 2407 (22.9%) |
| **Chronic disease present at cancer diagnosis** | AMI | 131 (0.4%) | 41 (0.4%) |
|  | Arrhythmia | 2529 (7.1%) | 706 (6.7%) |
|  | Asthma | 4698 (13.2%) | 1380 (13.1%) |
|  | CHF | 1948 (5.5%) | 529 (5%) |
|  | COPD | 3151 (8.8%) | 912 (8.7%) |
|  | Coronary | 5060 (14.2%) | 1465 (14%) |
|  | Dementia | 578 (1.6%) | 160 (1.5%) |
|  | Diabetes | 7802 (21.9%) | 2269 (21.6%) |
|  | Hypertension | 18456 (51.8%) | 5364 (51.1%) |
|  | IBD | 222 (0.6%) | 75 (0.7%) |
|  | Mental Health | 1301 (3.7%) | 349 (3.3%) |
|  | Mood Disorder | 4461 (12.5%) | 1302 (12.4%) |
|  | Osteoarthritis | 14887 (41.8%) | 4496 (42.8%) |
|  | Osteoporosis | 1932 (5.4%) | 555 (5.3%) |
|  | Renal Disease | 1621 (4.6%) | 433 (4.1%) |
|  | Rheumatoid Arthritis | 659 (1.9%) | 207 (2%) |
|  | Stroke | 953 (2.7%) | 258 (2.5%) |
| **Chemotherapy within 3 months after diagnosis** | Yes | 12003 (33.7%) | 3538 (33.7%) |
| **Radiation within 3 months after diagnosis** | Yes | 9475 (26.6%) | 2755 (26.2%) |
| **Cancer surgery within 3 months after diagnosis** | Yes | 17651 (49.6%) | 5199 (49.5%) |
| **Functional score within 3 months after diagnosis** | 0 | 9437 (26.5%) | 2725 (26%) |
|  | 1 | 5992 (16.8%) | 1796 (17.1%) |
|  | 2 | 1955 (5.5%) | 582 (5.5%) |
|  | 3 | 1032 (2.9%) | 295 (2.8%) |
|  | 4 | 330 (0.9%) | 91 (0.9%) |
|  | Missing | 16860 (47.4%) | 5009 (47.7%) |
| **Pain score within 3 months after diagnosis** | 0 | 11651 (32.7%) | 3515 (33.5%) |
|  | 1 | 6694 (18.8%) | 1963 (18.7%) |
|  | 2 | 3916 (11%) | 1103 (10.5%) |
|  | 3 | 1892 (5.3%) | 532 (5.1%) |
|  | Missing | 11453 (32.2%) | 3385 (32.2%) |
| **Wellbeing score within 3 months after diagnosis** | 0 | 6404 (18%) | 1911 (18.2%) |
|  | 1 | 9800 (27.5%) | 2771 (26.4%) |
|  | 2 | 6195 (17.4%) | 1862 (17.7%) |
|  | 3 | 2399 (6.7%) | 751 (7.2%) |
|  | Missing | 10808 (30.4%) | 3203 (30.5%) |
| **Dyspnea score within 3 months after diagnosis** | 0 | 23034 (64.7%) | 6752 (64.3%) |
|  | 1 | 2006 (5.6%) | 613 (5.8%) |
|  | Missing | 10566 (29.7%) | 3133 (29.8%) |
| **Depression score within 3 months after diagnosis** | 0 | 19292 (54.2%) | 5717 (54.5%) |
|  | 1 | 4607 (12.9%) | 1328 (12.7%) |
|  | Missing | 11707 (32.9%) | 3453 (32.9%) |
| **ESAS score within 3 months after diagnosis Tiredness** | 1 | 6330 (17.8%) | 1857 (17.7%) |
|  | 2 | 7083 (19.9%) | 2073 (19.7%) |
|  | 3 | 5350 (15%) | 1625 (15.5%) |
|  | 4 | 2805 (7.9%) | 841 (8%) |
|  | Missing | 14038 (39.4%) | 4102 (39.1%) |
| **Drowsiness** | 1 | 12120 (34%) | 3592 (34.2%) |
|  | 2 | 5420 (15.2%) | 1606 (15.3%) |
|  | 3 | 2892 (8.1%) | 860 (8.2%) |
|  | 4 | 1361 (3.8%) | 403 (3.8%) |
|  | Missing | 13813 (38.8%) | 4037 (38.5%) |
| **Nausea** | 1 | 17632 (49.5%) | 5223 (49.8%) |
|  | 2 | 2777 (7.8%) | 842 (8%) |
|  | 3 | 1008 (2.8%) | 309 (2.9%) |
|  | 4 | 461 (1.3%) | 121 (1.2%) |
|  | Missing | 13728 (38.6%) | 4003 (38.1%) |

| **Appetite** | 1 | 11354 (31.9%) | 3280 (31.2%) |
| --- | --- | --- | --- |
|  | 2 | 4675 (13.1%) | 1452 (13.8%) |
|  | 3 | 3759 (10.6%) | 1093 (10.4%) |
|  | 4 | 1744 (4.9%) | 551 (5.2%) |
|  | Missing | 14074 (39.5%) | 4122 (39.3%) |
| **Anxiety** | 1 | 7083 (19.9%) | 2159 (20.6%) |
|  | 2 | 7336 (20.6%) | 2122 (20.2%) |
|  | 3 | 4568 (12.8%) | 1299 (12.4%) |
|  | 4 | 2455 (6.9%) | 754 (7.2%) |
|  | Missing | 14164 (39.8%) | 4164 (39.7%) |
| **Has primary care physician** | Yes | 34790 (97.7%) | 10253 (97.7%) |
| **Hospitalization within 3 months after diagnosis** | Yes | 2187 (6.1%) | 648 (6.2%) |
| **Has a live-in caregiver** | No | 693 (1.9%) | 185 (1.8%) |
|  | Yes | 1582 (4.4%) | 471 (4.5%) |
|  | Missing or NA | 33331 (93.6%) | 9842 (93.7%) |
| **Received end of life care** | Yes | 1563 (4.4%) | 412 (3.9%) |

Median and IQR provided for continuous covariates; frequencies and percentages provided for binary or categorical covariates.
